# Sympathoadrenal Activation and Endothelial Damage Are Inter Correlated and Predict Increased Mortality in Patients Resuscitated after Out-Of-Hospital Cardiac Arrest. A Post Hoc Sub-Study of Patients from the TTM-Trial

**DOI:** 10.1371/journal.pone.0120914

**Published:** 2015-03-19

**Authors:** Pär I. Johansson, John Bro-Jeppesen, Jesper Kjaergaard, Michael Wanscher, Christian Hassager, Sisse R. Ostrowski

**Affiliations:** 1 Section for Transfusion Medicine Capital Region Blood Bank, Rigshospitalet, Copenhagen, Capital Region, Denmark; 2 Department of Surgery and Division of Acute Care Surgery, Centre for Translational Injury Research (CeTIR) at University of Texas Medical School at Houston, Houston, Texas, United States; 3 Department of Cardiology, Rigshospitalet, Copenhagen, Capital Region, Denmark; 4 Department of Cardiothoracic Anesthesiology The Heart Center, Rigshospitalet, Copenhagen, Capital Region, Denmark; Azienda Ospedaliero-Universitaria Careggi, ITALY

## Abstract

**Objective:**

Sympathoadrenal activation and endothelial damage are hallmarks of acute critical illness. This study investigated their association and predictive value in patients resuscitated from out-of-hospital cardiac arrest (OHCA).

**Methods:**

Post-hoc analysis of patients included at a single site in The Targeted Temperature Management at 33 degrees versus 36 degrees after Cardiac Arrest (TTM) trial. The main study reported similar outcomes with targeting 33 versus 36 degrees. TTM main study ClinicalTrials.gov: NCT01020916. One hundred sixty three patients resuscitated from OHCA were included at a single site ICU. Blood was sampled a median 135 min (Inter Quartile Range (IQR) 103-169) after OHCA. Plasma catecholamines (adrenaline, noradrenaline) and serum endothelial biomarkers (syndecan-1, thrombomodulin, sE-selectin, sVE-cadherin) were measured at admission (immediately after randomization). We had access to data on demography, medical history, characteristics of the OHCA, patients and 180-day outcome.

**Results:**

Adrenaline and noradrenaline correlated positively with syndecan-1 and thrombomodulin i.e., biomarkers reflecting endothelial damage (both p<0.05). Overall 180-day mortality was 35%. By Cox analyses, plasma adrenaline, serum sE-selectin, reflecting endothelial cell activation, and thrombomodulin levels predicted mortality. However, thrombomodulin was the only biomarker independently associated with mortality after adjusting for gender, age, rhythm (shockable vs. non-shockable), OHCA to return of spontaneous circulation (ROSC) time, shock at admission and ST elevation myocardial infarction (30-day Hazards Ratio 1.71 (IQR 1.05-2.77), p=0.031 and 180-day Hazards Ratio 1.65 (IQR 1.03-2.65), p=0.037 for 2-fold higher thrombomodulin levels).

**Conclusions:**

Circulating catecholamines and endothelial damage were intercorrelated and predicted increased mortality. Interventions aiming at protecting and/or restoring the endothelium may be beneficial in OHCA patients.

## Introduction

Out-of-hospital cardiac arrest (OHCA) is a leading cause of death among adults in the developed world. Despite state-of-the-art treatment from the earliest prehospital phase to hospital discharge, less than 30% survive, and many with poor functional outcome [1, 2]. Although most patients die in the earliest phase due to absent return of spontaneous circulation (ROSC), patients admitted to the hospital and intensive care unit (ICU) still face high mortality rates or survive with significant disabilities [1, 2]. One reason for the poor in-hospital outcome is the development of Post-Cardiac Arrest Syndrome (PCAS), characterized by varying degrees of 1) anoxic brain injury, 2) arrest-related myocardial dysfunction, 3) systemic ischemia/reperfusion injury and 4) persistent precipitating pathology i.e., the cause of cardiac arrest [3, 4]. PCAS results from a pathophysiologic process driven by a whole-body ischemia/reperfusion response, which triggers immediate and excessive activation of the inflammatory and hemostatic systems, leading to a sepsis-like syndrome [5] with ultimate development of (multiple) organ failure [3, 4]. Thus, similar to sepsis [6], OHCA patients present with excessive endothelial damage from the earliest phase of resuscitation [7–9].

Microcirculatory failure is a hallmark of acute critical illness: It is caused by numerous injurious hits on the vascular system, including the endothelium, and it is a driver of organ failure and thereby closely linked to outcome [6]. One of the hits encountered by the vascular system and endothelium in acute critical illness, including cardiac arrest, is a toxic high level of catecholamines [10–13], either endogenously released following excessive sympathoadrenal activation [14, 15] and/or exogenously administered as vasopressor/inotropic therapy [16, 17]. We have previously demonstrated associations between- and negative predictive values of high circulating catecholamines and endothelial damage in trauma [18, 19], sepsis [20] and ST segment elevation myocardial infarction (STEMI) [21] patients. However, no studies have previously investigated the association between circulating catecholamines, endothelial damage and outcome in cardiac arrest patients.

The objective of the present study was to investigate the association between sympathoadrenal activation, endothelial damage and outcome in OHCA patients, hypothesizing that excessive sympathoadrenal activation and endothelial damage would be associated and linked to poor outcome. We had access to previously collected plasma samples from OHCA patients included at a single site (Copenhagen, Denmark) in The Targeted Temperature Management at 33 degrees C versus 36 degrees C after Cardiac Arrest (TTM) trial [22]. The main TTM study reported similar outcomes with targeting 33 versus 36 degrees [22].

## Materials and Methods

### Trial protocol and patients

The present study represents a post hoc sub-study of patients from TTM trial (ClinicalTrials.gov number NCT01020916), a randomized clinical trial recruiting patients in 36 intensive care units (ICUs) in Europe and Australia [22]. The present sub-study was planned after conduct of the TTM-trial and after disclosure of the database from the TTM-trial.

The TTM protocol was approved by the ethics committees in each participating country and institution (in Denmark by the regional ethical committee (H-1–2010–059) and the Danish Data Protection Agency) and conducted in accordance with the Declaration of Helsinki. Written informed consent was obtained from a legal surrogate and from all patients who regained mental capacity [22]. Participant consent was documented as a signed consent form that was kept according to Danish Legislation. The procedure for obtaining written informed consent was approved by the ethics committee. The TTM inclusion criteria were patients ≥18 years of age who were unconscious (Glasgow Coma Scale (GCS) <8) on admission to the hospital after OHCA of presumed cardiac cause, irrespective of the initial rhythm. Eligible patients had more than 20 consecutive minutes of spontaneous circulation after resuscitation. The main exclusion criteria were an interval from the ROSC to screening of more than 240 minutes, unwitnessed arrest with asystole as the initial rhythm, suspected or known acute intracranial hemorrhage or stroke and a body temperature of less than 30°C [22].

Eligible patients for the present study were patients included at Rigshospitalet, Copenhagen University Hospital, Denmark (in total n = 171). Furthermore, to be included, an adequate volume of stored plasma and serum should be available from the pre-intervention admission blood sample to allow for investigation of the planned biomarkers (fulfilled in n = 163 as eight patients had too little sample volume to perform the planned biomarker analyses). The present study is based on these 163 patients.

We had access to the following data as part of the TTM trial protocol: Demography, medical history, characteristics of the cardiac arrest, patient characteristics at admission and outcome (mortality, Cerebral Performance Category (CPC) and modified Rankin scale (mRS); all surviving patients were followed until 180 days after the enrollment of the last patient) [22].

### Blood samples

Blood was sampled from an arterial line within 5 minutes following inclusion and randomization in the study. The sample was divided for blood gas analysis (ABG, Radiometer ABL 725/735, Copenhagen, Denmark), routine biochemistry and research analyses (citrate, heparin and EDTA plasma, serum). All samples were centrifuged immediately at 4°C. Plasma and serum aliquots were stored at -80°C until thawed for analysis.

### Enzyme linked immunosorbent assay (ELISA) analyses

Biomarkers of sympathoadrenal activation (adrenaline, noradrenaline) and endothelial glycocalyx damage (syndecan-1) [23], endothelial cell activation (sE-selectin) [24, 25], endothelial cell injury (soluble thrombomodulin) [24–26] and endothelial junction disruption (sVE-cadherin) [27] were measured in uniplicate by commercially available immunoassays in EDTA plasma (catecholamines) and serum (endothelial biomarkers) according to the manufactures recommendations: Plasma (p)-adrenaline and p-noradrenaline (2-CAT ELISA^FAST TRACK^, Labor Diagnostica Nord GmbH & Co. KG, Nordhorn, Germany; lower limit of detection (LLD) 10 pg/ml (adrenaline) and 50 pg/ml (noradrenaline), respectively); syndecan-1 (Diaclone, Nordic Biosite, Copenhagen, Denmark; LLD 4.94 ng/ml); sE-selectin (R&D Systems Europe, Ltd., Abingdon, UK; LLD 0.009 ng/ml); thrombomodulin (Diaclone, Nordic Biosite, Copenhagen, Denmark; LLD 0.31 ng/ml) and sVE-cadherin (R&D Systems Europe, Ltd., Abingdon, UK; LLD 0.113 ng/ml). Values below LLD were recorded as the LLD value (n = 5 for adrenaline, n = 3 for noradrenaline, none for the remaining biomarkers).

### Statistics

Statistical analysis was performed using SAS 9.1.3 SP4 (SAS Institute Inc., Cary, NC, US).

Patients stratified according to shockable rhythm (yes vs. no, see Table 1 legend for definition) or high vs. low thrombomodulin level (high: >median vs. low: ≤median) were compared by Mann-Whitney U test or Chi-square/Fisher´s exact tests, as appropriate.

**Table 1:** Demography, medical history, characteristics of the cardiac arrest, patient admission characteristics and outcome in all patients (n = 163) and in patients stratified according to admission serum thrombomodulin (high (>median) vs. low (≤median), n = 160) admitted to a tertiary university hospital after out-of-hospital cardiac arrest (OHCA).

		All patients	High thrombomodulin	Low thrombomodulin	p-value
		(n = 163)	(n = 78)	(n = 82)	
**Demography**					
Age	years	62 (53–68)	64 (58–69)	60 (51–66.75)	**0.018**
Male gender	n (%)	143 (88%)	76 (97%)	64 (78%)	**<0.001**
Body Mass Index	kg/m^2^	25.4 (23.5–27.8)	25.0 (23.5–27.8)	25.4 (23.6–29.3)	NS
**Medical history**					
Chronic heart failure	n (%)	6 (3.7%)	2 (2.6%)	3 (3.7%)	NS
Previous AMI	n (%)	24 (14.7%)	11 (14.1%)	11 (13.4%)	NS
Ischemic heart disease	n (%)	33 (20.4%)	16 (20.8%)	15 (18.3%)	NS
Previous cardiac arrhythmia	n (%)	20 (12.3%)	12 (15.4%)	7 (8.5%)	NS
Arterial hypertension	n (%)	50 (30.7%)	22 (28.2%)	25 (30.5%)	NS
Previous TIA or stroke	n (%)	10 (6.1%)	4 (5.1%)	5 (6.1%)	NS
Diabetes mellitus	n (%)	22 (13.5%)	12 (15.4%)	10 (12.2%)	NS
Asthma or COPD	n (%)	4 (2.5%)	2 (2.6%)	2 (2.4%)	NS
Previous PCI	n (%)	12 (7.4%)	5 (6.4%)	5 (6.1%)	NS
Previous CABG	n (%)	7 (4.3%)	3 (3.8%)	3 (3.7%)	NS
Pacemaker	n (%)	3 (1.8%)	1 (1.3%)	1 (1.2%)	NS
**Characteristics of the cardiac arrest**					
Location (R / P / O)	%	56%/43%/1%	56%/44%/0%	56%/42%/2%	NS
Bystander witnessed arrest	n (%)	146 (89.6%)	69 (88.5%)	74 (90.2%)	NS
Bystander CPR	n (%)	129 (79.1%)	62 (79.5%)	64 (78%)	NS
Shockable rhythm	n (%)	145 (89.0%)	68 (87.2%)	75 (91.5%)	NS
Adrenaline administration	n (%)	122 (74.8%)	63 (80.8%)	56 (68.3%)	0.071
Dose of adrenaline	mg	2 (1–4)	2 (1–4)	1 (0–3)	**0.017**
Pre-hospital intubation	n (%)	128 (79.0%)	57 (74.0%)	68 (82.9%)	NS
OHCA to ROSC	min	23 (14–30)	24 (14–30)	20 (14–32)	NS
**Patient characteristics on admission**					
Cormeal reflex	n (%)	157 (96.3%)	75 (96.2%)	79 (96.3%)	NS
Pupil reflex	n (%)	150 (92.0%)	70 (89.7%)	77 (93.9%)	NS
pH	-Log[H^+^]	7.2 (7.1–7.3)	7.1 (7.0–7.2)	7.2 (7.1–7.3)	**0.001**
Lactate	ng/ml	7.0 (3.8–11.0)	8.4 (4.4–12.5)	5.0 (3.0–9.3)	**0.010**
Shock on admission	n (%)	17 (10.4%)	11 (14.1%)	6 (7.3%)	NS
Initial temperature	°C	35.5 (35.0–36.0)	35.6 (34.7–36.0)	35.5 (35.2–36.0)	NS
ECG findings (U / S / L / A / O)	%	25%/58%/7%/1%/9%	27%/59%/4%/0%/10%	23%/59%/10%/0%/8%	NS
**Biomarker levels on admission**					
OHCA to randomization	min	135 (103–169)	142 (104–169)	130 (102–169)	NS
Adrenaline	pg/ml	542 (108–1,128)	792 (215–1,865)	368 (95–1,025)	**0.041**
Noradrenaline	pg/ml	698 (389–1,542)	1,090 (439–2,117)	519 (308–1,206)	**0.004**
Syndecan-1	ng/ml	152 (74–235)	161 (86–248)	147 (71–224)	NS
Thrombomodulin	ng/ml	7.0 (5.3–9.2)	9.3 (7.7–11.6)	5.4 (4.7–6.2)	NA
sE-selectin	ng/ml	34 (27–45)	37 (28–45)	33 (25–44)	NS
sVE-cadherin	ng/ml	2,595 (2,303–3,174)	2,818 (2,359–3,286)	2,516 (2,185–3,019)	**0.016**
**Outcome**					
Discharge facility (O / R / H)	%	66%/4%/30%	76%/2%/22%	60%/5%/35%	NS
180-day CPC 1–2	n (%)	101 (62%)	40 (51%)	59 (72%)	**0.007**
180-day mRS 0–3	n (%)	102 (63%)	41 (53%)	59 (72%)	**0.001**
7-day mortality	n (%)	33 (20.2%)	20 (25.6%)	13 (15.9%)	0.126
30-day mortality	n (%)	53 (32.5%)	34 (43.6%)	18 (22.0%)	**0.004**
180-day mortality	n (%)	57 (35.0%)	36 (46.2%)	20 (24.4%)	**0.004**

Data are presented as medians (IQR) or n (%). Patients stratified according to the median serum level of thrombomodulin at admission were compared by Mann-Whitney U test or Chi-square/Fisher´s exact tests as appropriate, with p-values <0.05 shown in bold. AMI, acute myocardial infarction. TIA, transient ischemic attack. COPD, chronic obstructive pulmonary disease. PCI, percutaneous coronary intervention. CABG, coronary artery bypass graft. Location: R, place of residence; P, public place; O, other. CPR, cardio-pulmonary resuscitation. Shockable rhythm: ventricular fibrillation, nonperfusing ventricular tachycardia, unknown rhythm responsive to shock, perfusing rhythm after bystander-initiated defibrillation; non-shockable rhythm: asystole, pulseless electrical activity, unknown rhythm not responsive to shock. ROSC, return of spontaneous circulation. ECG (electrocardiography) findings: U, unchanged from previously/normal; S, ST-segment myocardial infarction (STEMI); L, left bundle branch block; A, atrial fibrillation or flutter; O, other. Discharge facility: O, other hospital/intensive care unit; R, rehabilitation facility; H, home; CPC, Cerebral Performance Category (1–2 designates good outcome); mRS, modified Rankin Scale (0–3 designates good outcome).

Simple correlations were investigated by Spearman´s correlations with results displayed as rho and p-values. To investigate the contribution of p-catecholamines, individual biomarkers of endothelial activation or damage, characteristics of the OHCA and demographic variables to the variation in levels of the endothelial biomarkers (syndecan-1, thrombomodulin, sE-selectin and sVE-cadherin), multivariate backwards linear regression analysis were performed including variables that were either found to correlate with or expected to influence the investigated biomarkers: Age, BMI, number of defibrillations, time from OHCA to ROSC, pH, p-adrenaline, p-noradrenaline, syndecan-1, sE-selectin, thrombomodulin, sVE-cadherin and ECG finding at admission (STEMI vs. other). Results are presented as regression coefficients (β) with 95% confidence intervals (CI), p-values and adjusted R^2^.

The predictive value of admission p-catecholamines (dichotomized by the median: high vs. low) and biomarkers of endothelial activation and damage (log_2_ transformed) for 7-day, 30-day and 180-day mortality was investigated by univariate and multivariate Cox proportional-hazards models, the latter after adjusting for previously described [22] predictive variables: Age, gender, rhythm (shockable vs. non-shockable), time from OHCA to ROSC, shock at admission and admission ECG finding (STEMI vs. other). Results are presented as hazards ratio (HR) with 95% CI and p-values. Furthermore, the predictive value of high vs. low p-catecholamines and biomarker levels (dichotomized by the median: high vs. low) for mortality were investigated by Log-Rank tests based on Kaplan-Meier survival curves in patients stratified according to these variables. Results are presented with χ^2^ and p-values.

Descriptive data are presented as medians with inter quartile ranges (IQR) or as n (proportions). P-values <0.05 were considered significant.

## Results

### Patients

The 163 patients had a median age of 62 years, were predominantly men (88%) and had an overall 7-, 30- and 180-day mortality rate of 20%, 33% and 35%, respectively. A comparable number of patients received the 33 and 36°C temperature intervention (n = 81 vs. n = 82, respectively). A detailed description of patient demography, medical history, admission physiology, outcome and characteristics of the cardiac arrest is provided in Table 1.

### Factors associated with p-catecholamines and biomarkers of endothelial activation and damage

Levels of circulating catecholamines and endothelial derived biomarkers are displayed in Table 1.

#### Demography and medical history

P-catecholamines were neither associated with age nor gender (data not shown). Syndecan-1 correlated negatively with age (rho = -0.16, p = 0.048) whereas thrombomodulin correlated positively with age (rho = 0.22, p = 0.006) and was higher in men (7.3 vs. 5.2 ng/ml, p<0.001). sE-selectin correlated with BMI (rho = 0.23, p = 0.003) and was higher in patients with diabetes (41 vs. 33 ng/ml, p = 0.028).

#### Inter-correlations p-catecholamine and endothelial biomarker levels

**Fig 1:**
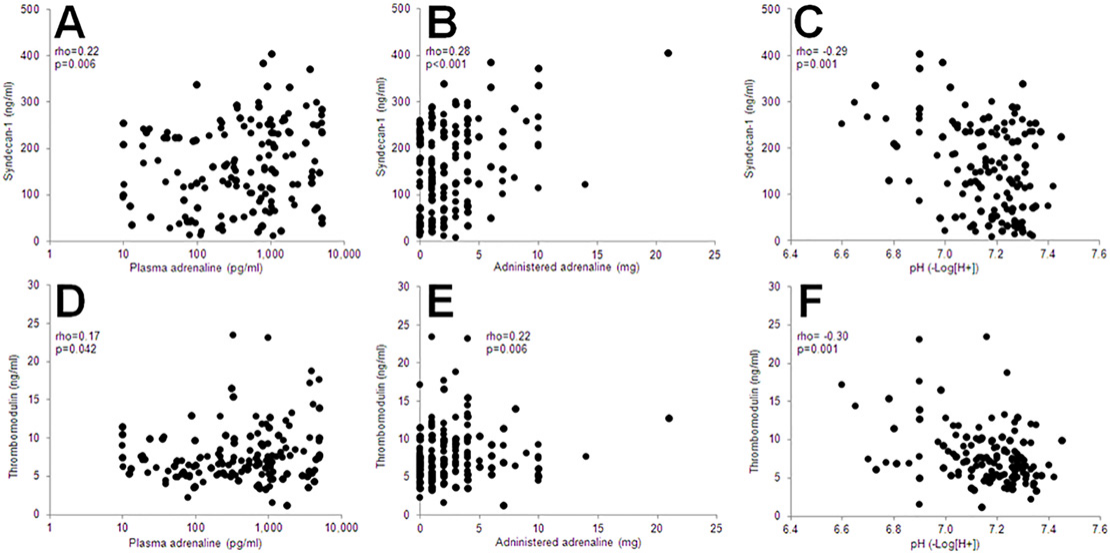
Correlations between admission levels of syndecan-1 or thrombomodulin, reflecting endothelial glycocalyx and cell damage, respectively, and plasma (p)-adrenaline (pg/ml) (A and D), administered adrenaline (mg) (B and E) and pH (C and F) in 163 OHCA patients P- and rho-values for Spearman´s correlations are displayed.

Admission levels of both p-adrenaline and p-noradrenaline were positively inter correlated (rho = 0.27, p = 0.001) and correlated with syndecan-1 (Fig. 1A and rho = 0.19, p = 0.022) and thrombomodulin (Fig. 1D and rho = 0.24, p = 0.003).

Among the endothelial biomarkers, sVE-cadherin correlated with thrombomodulin (rho = 0.23, p = 0.003) and sE-selectin (rho = 0.20, p = 0.014).

#### Characteristics of the OHCA and admission variables

Syndecan-1 correlated with the administered adrenaline dose, pH (Fig. 1BC), lactate (rho = 0.35, p<0.001), time from OHCA to ROSC (rho = 0.31, p<0.001) and number of defibrillations (rho = 0.25, p = 0.002) and thrombomodulin correlated with administered adrenaline dose, pH (Fig. 1EF) and lactate (rho = 0.25, p = 0.004).

Patients with STEMI-induced OHCA (n = 95, 58%) had almost 3-fold higher syndecan-1 levels compared to patients with other causes of OHCA (median 206 ng/ml (IQR 125–249) vs. 81 ng/ml (IQR 39–167), p<0.001). STEMI OHCA patients received a prehospital bolus administration of unfractionated heparin (10,000 IE) and a higher proportion of STEMI OHCA patients received adrenaline during resuscitation (n = 77 (81%) vs. n = 45 (66%), p = 0.031). Despite the higher adrenaline administration in STEMI OHCA patients, p-catecholamine levels were comparable in STEMI OHCA and other OHCA patients as well as sE-selectin and thrombomodulin levels (data not shown). sVE-cadherin was approximately 10% lower in STEMI OHCA patients (median 2,558 ng/ml (IQR 2,157–3,030) vs. 2,763 ng/ml (IQR 2405–3,294), p = 0.024). Survival was comparable in STEMI and other OHCA patients (data not shown).

The level of circulating catecholamines and endothelial biomarkers was comparable in patients stratified according to shockable (n = 145) vs. non-shockable rhythm (n = 18) (data not shown).

### Factors independently associated with endothelial activation and damage

Multivariate linear regression analysis revealed that STEMI as cause of the OHCA, increased time from OHCA to ROSC and lower pH were independently associated with higher syndecan-1 (glycocalyx damage), explaining 36% of its variation (Table 2). Likewise, lower age, higher BMI and higher sVE-cadherin were independently associated with higher sE-selectin (endothelial activation); higher age, lower pH and higher sVE-cadherin were independently associated with higher thrombomodulin (endothelial cell injury) and higher thrombomodulin and higher sE-selectin were independently associated with higher sVE-cadherin (endothelial junction disruption) (Table 2).

**Table 2:** Variables independently associated with admission biomarkers reflecting endothelial glycocalyx and cell activation and/or damage, and endothelial cell junction function (syndecan-1, sE-selectin, thrombomodulin and sVE-cadherin, respectively) by backwards multivariate linear regression analysis in 163 patients admitted to a tertiary university hospital after out-of-hospital cardiac arrest.

		Syndecan-1		sE-selectin		Thrombomodulin		sVE-cadherin	
		Adj. R^2^ = 0.36		Adj. R^2^ = 0.14		Adj. R^2^ = 0.24		Adj. R^2^ = 0.13	
		β (95%CI)	p	β (95%CI)	p	β (95%CI)	p	β (95%CI)	p
Age	years		NS	-0.23 (-0.45- -0.02)	**0.034**	0.09 (0.04–0.14)	**0.001**		NS
BMI	kg/m^2^		NS	0.94 (0.31–1.57)	**0.004**		NS		NS
OHCA to ROSC	min	1.31 (0.48–2.13)	**0.002**		NS		NS		NS
pH	-log[H^+^]	-119 (-216- -21)	**0.018**		NS	-6.8 (-10.5- -3.1)	**<0.001**		NS
STEMI	Yes	84 (56–111)	**<0.001**		NS		NS		NS
Syndecan-1	2-fold	NA	NA		NS		NS		NS
sE-selectin	2-fold		NS	NA	NA		NS	251 (21–481)	**0.033**
Thrombomodulin	2-fold		NS		NS	NA	NA	333 (146–519)	**<0.001**
sVE-cadherin	2-fold		NS	9.9 (3.6–16.3)	**0.002**	2.6 (1.0–4.2)	**0.002**	NA	NA

Regression coefficients (β) with 95% confidence intervals (95%CI), p-values and adjusted R^2^ are displayed, with p-values <0.05 shown in bold. Predicted changes in syndecan-1 (ng/ml, reflecting glycocalyx damage), sE-selectin (ng/ml, reflecting endothelial activation), Thrombomodulin (ng/ml, reflecting endothelial cell injury) and sVE-cadherin (ng/ml, reflecting endothelial junction disruption) associated with one unit increase in the explanatory variables (age (1 year older), BMI, number of defibrillations (NS all over, data not shown), time from OHCA to ROSC (min), pH, STEMI (yes), p-adrenaline and p-noradrenaline (10-fold higher, NS all over, data not shown), syndecan-1, thrombomodulin, sE-selectin and VE-cadherin (all 2-fold higher). NS, non-significant. NA, non-applicable.

### Sympathoadrenal activation, endothelial damage and outcome

**Fig 2:**
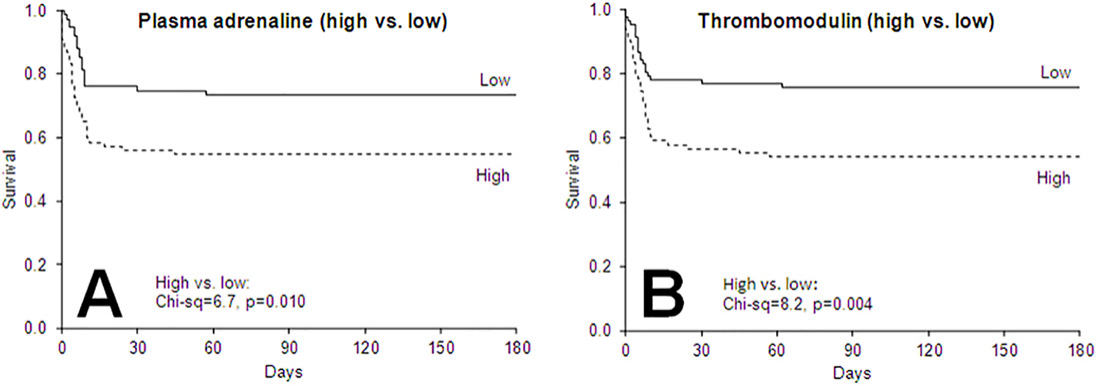
Kaplan-Meier plots displaying 180-day mortality in 163 OHCA patients stratified according to median levels (high vs. low) of A) plasma (p)-adrenaline and B) Serum thrombomodulin at hospital admission. Chi-square and p-values for log-rank tests are shown.

Log-Rank tests based on Kaplan-Meier survival curves for medians (high vs. low) of admission biomarker levels revealed that high p-adrenaline and high thrombomodulin were associated with increased 30-day (p = 0.006 and p = 0.004, respectively) and 180-day mortality (Fig. 2AB). High p-adrenaline was the only investigated biomarker associated with higher 7-day mortality (p = 0.005).

By Cox proportional-hazards analyses, high p-adrenaline (above median) was a univariate predictor of increased mortality (180-day: HR 2.0 (95%CI 1.7–3.5), χ^2^ = 6.3, p = 0.012; 30-day: HR 2.2 (95%CI 1.2–3.8), χ^2^ = 7.0, p = 0.008; 7-day: HR 2.8 (95%CI 1.3–6.1), χ^2^ = 6.9, p = 0.008). Furthermore, thrombomodulin, sE-selectin, age, rhythm and time from OHCA to ROSC were univariate predictors of mortality (Table 3). In the adjusted models, higher thrombomodulin remained an independent predictor of higher 30-day and 180-day mortality together with higher age, non-shockable rhythm and increased time from OHCA to ROSC (Table 3).

**Table 3:** Cox Proportional Hazards models predicting 7-day, 30-day and 180-day mortality in 163 patients admitted to a tertiary university hospital after out-of-hospital cardiac arrest.

		Univariate		Syndecan-1		sE-selectin		Thrombomodulin		sVE-cadherin	
				(2-fold higher)		(2-fold higher)		(2-fold higher)		(2-fold higher)	
		HR (95%CI)	p	HR (95%CI)	p	HR (95%CI)	p	HR (95%CI)	p	HR (95%CI)	p
**7-day mortality**	univariate	-	-	1.29 (0.93–1.81)	0.126	0.51 (0.28–0.94)	**0.031**	2.09 (1.20–3.66)	**0.010**	0.84 (0.36–1.97)	NS
	multivariate	-	-	1.13 (0.79–1.62)	NS	0.58 (0.31–1.10)	0.094	1.34 (0.74–2.44)	NS	0.79 (0.34–1.86)	NS
Gender	male	1.01 (0.35–2.87)	NS	1.29 (0.40–4.12)	NS	1.23 (0.39–3.83)	NS	1.08 (0.32–3.64)	NS	1.29 (0.41–4.06)	NS
Age	1 year older	1.05 (1.02–1.08)	**0.003**	1.07 (1.03–1.11)	**<0.001**	1.07 (1.02–1.10)	**0.002**	1.06 (1.02–1.10)	**0.022**	1.07 (1.03–1.11)	**<0.001**
Shockable rhythm	yes	0.22 (0.10–0.46)	**<0.001**	0.21 (0.10–0.45)	**<0.001**	0.19 (0.08–0.41)	**<0.001**	0.22 (0.10–0.49)	**<0.001**	0.20 (0.09–0.44)	**<0.001**
OHCA to ROSC	1 min longer	1.02 (1.01–1.03)	**0.002**	1.01 (1.00–1.03)	0.065	1.02 (1.01–1.04)	**0.009**	1.02 (1.00–1.03)	**0.028**	1.02 (1.01–1.03)	**0.022**
Shock at admission	yes	2.32 (0.96–5.61)	0.063	1.36 (0.49–3.81)	NS	0.92 (0.30–2.83)	NS	1.25 (0.44–3.56)	NS	1.23 (0.43–3.52)	NS
STEMI	yes	1.72 (0.82–3.62)	0.151	2.22 (0.97–5.09)	0.060	2.35 (1.01–5.46)	**0.047**	2.44 (1.09–5.47)	**0.031**	2.23 (0.96–5.17)	0.062
**30-day mortality**	univariate	-	-	1.27 (0.98–1.65)	0.072	0.69 (0.42–1.12)	0.135	2.43 (1.56–3.79)	**<0.001**	1.33 (0.65–2.72)	NS
	multivariate	-	-	1.15 (0.87–1.53)	NS	0.68 (0.42–1.12)	0.129	1.71 (1.05–2.77)	**0.031**	1.05 (0.53–2.11)	NS
Gender	male	0.91 (0.41–2.02)	NS	1.11 (0.45–2.73)	NS	1.08 (0.45–2.61)	NS	0.80 (0.31–2.05)	NS	1.12 (0.45–2.77)	NS
Age	1 year older	1.05 (1.02–1.07)	**<0.001**	1.07 (1.04–1.10)	**<0.001**	1.06 (1.03–1.09)	**<0.001**	1.05 (1.02–1.09)	**<0.001**	1.06 (1.03–1.10)	**<0.001**
Shockable rhythm	yes	0.25 (0.13–0.46)	**<0.001**	0.25 (0.13–0.49)	**<0.001**	0.21 (0.10–0.42)	**<0.001**	0.28 (0.14–0.57)	**<0.001**	0.24 (0.12–0.47)	**<0.001**
OHCA to ROSC	1 min longer	1.02 (1.01–1.03)	**<0.001**	1.02 (1.01–1.03)	**0.007**	1.02 (1.01–1.04)	**<0.001**	1.02 (1.01–1.03)	**0.002**	1.02 (1.01–1.03)	**0.002**
Shock at admission	yes	1.87 (0.88–3.97)	0.102	0.97 (0.38–2.44)	NS	0.73 (0.27–1.97)	NS	0.85 (0.33–2.18)	NS	0.96 (0.38–2.46)	NS
STEMI	yes	1.27 (0.73–2.21)	NS	1.72 (0.90–3.28)	0.099	1.88 (1.00–3.55)	0.051	1.91 (1.02–3.55)	**0.042**	1.91 (1.01–3.64)	**0.048**
**180-day mortality**	univariate	-	-	1.26 (0.98–1.62)	0.068	0.78 (0.48–1.24)	NS	2.40 (1.56–3.69)	**<0.001**	1.35 (0.68–2.69)	NS
	multivariate	-	-	1.16 (0.89–1.53)	NS	0.74 (0.46–1.18)	NS	1.65 (1.03–2.65)	**0.037**	1.02 (0.52–2.01)	NS
Gender	male	0.99 (0.49–2.18)	NS	1.21 (0.49–2.99)	NS	1.18 (0.49–2.87)	NS	0.90 (0.35–2.30)	NS	1.23 (0.50–3.03)	NS
Age	1 year older	1.05 (1.02–1.07)	**<0.001**	1.06 (1.04–1.09)	**<0.001**	1.06 (1.03–1.09)	**<0.001**	1.05 (1.02–1.08)	**<0.001**	0.23 (0.12–0.45)	**<0.001**
Shockable rhythm	yes	0.24 (0.13–0.43)	**<0.001**	0.24 (0.13–0.47)	**<0.001**	0.21 (0.11–0.40)	**<0.001**	0.28 (0.14–0.54)	**<0.001**	1.06 (1.03–1.09)	**<0.001**
OHCA to ROSC	1 min longer	1.02 (1.01–1.03)	**<0.001**	1.02 (1.01–1.03)	**0.005**	1.02 (1.01–1.04)	**<0.001**	1.02 (1.01–1.03)	**0.001**	1.02 (1.01–1.03)	**<0.001**
Shock at admission	yes	1.73 (0.82–3.64)	0.153	0.88 (0.35–2.20)	NS	0.67 (0.25–1.81)	NS	0.78 (0.31–1.99)	NS	0.87 (0.34–2.20)	NS
STEMI	yes	1.14 (0.67–1.93)	NS	1.44 (0.78–2.66)	NS	1.62 (0.89–2.94)	0.117	1.61 (0.90–2.90)	0.112	1.61 (0.87–2.96)	NS

Hazards ratios (HR) with 95% confidence intervals (HR (95% CI)) and p-values associated with one unit increases in age (1 year older), gender (being male), shockable rhythm (yes), time from OHCA to ROSC (1 minute longer), shock at admission (yes) and 2-fold increases (log_2_ transformed) in serum levels of syndecan-1, sE-selectin, thrombomodulin and sVE-cadherin. Only p-values <0.20 are shown, with p<0.05 shown in bold.

### Patients with high vs. low degree of endothelial cell injury

Since thrombomodulin i.e., endothelial cell injury, was an independent predictor of mortality, we compared patients with high vs. low thrombomodulin levels at admission (Table 1) revealing that patients with high thrombomodulin were older, predominantly men, received a higher adrenaline dose during resuscitation, had lower pH and higher lactate and had higher p-adrenaline and p-noradrenaline (both approximately 2-fold increased) and also higher sVE-cadherin (Table 1). Patients with high thrombomodulin levels also had a poorer outcome according to the Cerebral Performance Category (CPC) and modified Rankin scale (mRS) and they had higher mortality (Table 1).

## Discussion

In the present study, OHCA patients presented with high and inter-correlated levels of circulating catecholamines and biomarkers of excessive endothelial damage. Increased time from OHCA to ROSC, lower pH and STEMI were independently associated with higher syndecan-1 levels, a marker of glycocalyx damage, whereas lower pH, higher age and higher sVE-cadherin were independently associated with higher thrombomodulin levels, a marker of endothelial cell injury. By Cox proportional-hazards analyses, high thrombomodulin levels independently predicted 30-day and 180-day mortality.

Acute critical illness is accompanied by excessive sympathoadrenal activation that induces widespread, dose-dependent effects on the vascular system [10, 11, 14], including the endothelium [15]. We have proposed [11] that endogenously released catecholamines ensure oxygen supply to vital organs in acute critical illness by balancing the clotting ability of the circulating blood according to the degree of endothelial anti-/procoagulation in the microcirculation. Hereby, progressive endothelial damage (and procoagulation) in the microcirculation is balanced by increasing hypocoagulability (coagulopathy) in the circulating blood [11]. Given this, the evolutionary rational for the coagulopathy observed in many acute critically ill patients [28–30], may be that evolution has prioritized tissue oxygenation above hemostasis. We have reported of associations between- and negative predictive values of high circulating catecholamines and endothelial damage in trauma, sepsis and STEMI patients [18–21]. In line with this, the present study found that circulating levels of adrenaline and noradrenaline correlated positively with syndecan-1 and thrombomodulin levels, biomarkers of endothelial glycocalyx and cell damage, respectively [23, 24, 26]. Furthermore, high circulating thrombomodulin was an independent predictor of mortality. The present study thus supports the notion that different “injurious” hits can mount a similar, universal response, characterized by excessive sympathoadrenal activation, endothelial damage and coagulopathy.

Gando et al [7] suggested years ago that the endothelial damage observed in OHCA patients could in part be attributed to both exogenous administered and endogenously released catecholamines. Recently, Hagihara et al [16] reported in a prospective observational propensity score analysis of data from 417,188 OHCA patients that use of prehospital adrenaline was associated with increased chance of ROSC before hospital arrival but also with decreased chance of survival and good functional outcomes 1 month after the event. This is in accordance with previous studies also questioning the beneficial effect of adrenaline administration in CA [17, 31]. Interestingly, several retrospective studies of trauma-, sepsis- and surgical patients have reported a paradoxical survival benefits for patients receiving β-blockers as part of their regular medication at the time of the injurious hit [32–36], despite these patients often being older and suffering from more co-morbidities compared to patients not receiving β-blockers. Randomized clinical studies comparing β-blockers with placebo in trauma, sepsis and patients suffering from acute ischemic heart disease have confirmed these findings [37, 38]. It is tempting to speculate that the beneficial effects of β-blockers observed in many acute critically ill patients may in part be due to protective effects on the endothelium in conditions with excessive catecholamine release.

Glycocalyx damage is associated with pathophysiologic sequels like capillary leakage, accelerated inflammation, platelet activation and loss of vascular responsiveness [39, 40]. Previous studies investigating circulating syndecan-1 levels as a surrogate for endothelial glycocalyx damage have reported increased levels in trauma [18, 41], sepsis [20, 42], major vascular- [23] and abdominal surgery [42], STEMI [21] and OHCA [8] patients, with the highest levels in non-survivors and/or the most sick patients. In the present study, STEMI OHCA patients had several-fold higher syndecan-1 levels compared to patients with other causes of OHCA. It is not known if this is due to downstream effects of the STEMI, to iatrogenous factors such as the heparin IV injection given to these patients or to other factors. It is well described that heparin can destabilize the endothelial glycocalyx [43] through induction of a rapid dose-dependent release of glycocalyx adsorbed heparan sulphate-bound proteins [44, 45], and it is possible that this may contribute to accelerated glycocalyx damage following a “second hit” (e.g. ischemia/reperfusion) [23]. However, shocked STEMI patients without cardiac arrest who receive a comparable IV injection of heparin [21] display lower circulating syndecan-1 level compared to STEMI OHCA patients (median 129 ng/ml vs. 206 ng/ml), indicating that other factors than heparin i.e., exogenous adrenaline administration, defibrillations, time from OHCA to ROSC, pH and lactate (shock) (these were all correlated with syndecan-1 in the present study) contribute to the glycocalyx damage [8, 39].

There is emerging evidence that the change from a normal quiescent endothelium to endothelial cell activation, glycocalyx damage, junctional disruption and ultimate endothelial cell injury reflects a progression from reversible to irreversible endothelial damage [25, 41, 46]. In the present study, we investigated different endothelial derived molecules as surrogate markers for glycocalyx damage, endothelial cell activation, endothelial junction disruption and endothelial cell injury, to reveal the influence of progressive endothelial disruption on outcome in OHCA patients. Interestingly, we found that biomarkers reflecting endothelial damage (syndecan-1, thrombomodulin) were independently associated with shock degree (time from OHCA to ROSC, pH) whereas biomarkers reflecting endothelial activation (sE-selectin) and junctional disruption (sVE-cadherin) were independently associated with patient demography and/or other endothelial biomarkers. The finding that sE-selectin, sVE-cadherin and thrombomodulin were inter correlated supports the notion that endothelial activation, junctional disruption and cell injury are linked at the biologic level. Finally, we found that high circulating thrombomodulin was an independent predictor of increased mortality. In accordance with previous studies, higher age, male gender, high catecholamine levels and shock were all associated with high thrombomodulin levels [21]. The strong predictive value of thrombomodulin supports the notion that patients with the most severe form of endothelial injury have the poorest outcome. The finding that post-CA therapeutic hypothermia may attenuate the anoxic brain injury and improve outcome [47, 48], emphasizes that therapeutic interventions applied after ROSC can be beneficial. Given this, we infer that interventions aiming at protecting and/or restoring the endothelium, administered at the earliest possible, may be able to alleviate the downstream endothelial damage and the ensuing PCAS, and thereby improve patient outcome. A randomized clinical trial to test this hypothesis is currently underway.

The present study had several limitations. First, the observational nature of study does not allow independent evaluations of the cause-and-effect relationships suggested. Second, the IV heparin injection to STEMI OHCA patients makes it impossible to differentiate between potential effects of the STEMI itself vs. heparin on glycocalyx damage. Third, the study was a post hoc sub-study of the TTM trial, a randomized multicenter trial, where we only investigated patients included at a single site [49]. Given this, the present findings should be considered hypothesis-generating and the p-values as explorative in nature.

## Conclusions

In the present study, OHCA patients presented with evidence of excessive endothelial damage that correlated with circulating catecholamine levels, time from OHCA to ROSC and biochemical shock markers. High thrombomodulin levels, reflecting endothelial cell injury, independently predicted increased mortality. We speculate that interventions aiming at protecting and/or restoring the endothelium may be of value in OHCA patients.
